# Hemoptysis, a developing world perspective

**DOI:** 10.1186/1471-2466-6-1

**Published:** 2006-01-13

**Authors:** Omer Ashraf

**Affiliations:** 1 Medical College, Aga Khan University Hospital, Stadium Road, Karachi, 74800, Pakistan

## Abstract

**Background:**

Hemoptysis is a significant clinical presentation in respiratory medicine. Often a life threatening emergency, it mandates prompt assessment and intervention. Various investigations and management protocols are proposed globally, to advocate a standardized approach towards patients presenting with hemoptysis. It is the etiology, however, that has been known to influence clinical outcome and prognosis. With marked contrast in geographical patterns of pulmonary pathologies, etiological agents for hemoptysis vary over the world. Studies in West, usually demonstrate neoplastic and non-granulomatous causes to be the leading agents for hemoptysis. The diagnostic accuracy of various investigations and efficacy of management alternatives has been established there. Developing nations differ in their burden of diseases of lung. Lack of health resources and initiative often prevent quality research in critical areas.

**Design:**

This is a retrospective observational study with a cross-sectional design in which charts of all patients admitted with the presentation of haemoptysis in the past ten years will be reviewed, at Aga Khan University Hospital, Karachi, Pakistan. A series of variables, based on previous literature on haemoptysis related to the objectives of present study, will be determined in the study. Demographics, co-morbids and etiology will be determined. Findings of various investigation modalities and their accuracy in localizing the bleeding site will be determined. Efficacy of different management strategies will also be observed. Also observed will be any complications and follow-up.

**Discussion:**

Pakistan is a third world nation of over 150 million, established as highly endemic for pulmonary tuberculosis. To date no study has been generated to look into hemoptysis patterns, in this nation. Lack of evidence based medicine poses a major hindrance towards confident decision-making in the approach towards a patient presenting with hemoptysis in this country. This study is devised to obtain the first insight in this direction, from this part of the world. The etiologies, accuracy of various investigations and efficacy of treatment options will be investigated. The results and conclusions will prove to be of value not just for health administrators in this country, but many other regions that share similarities in patterns of pulmonary pathologies.

## Background

Hemoptysis refers to expectoration of blood from respiratory tract. Often lethal, it is a frightening presentation for patient and relatives. A major emergency in pulmonary medicine, it necessitates a timely and apt approach.

While emergency resuscitation protocols may overlap, medical advance is usually directed on basis of severity and etiology of hemoptysis. Apposite clinical assessment and investigations are thus paramount in formulating the management proposal. After adequate airway protection and insurance of ventilatory and cardiovascular stability, in case of gross hemoptysis, imaging is usually resorted to, for indication of the pathology and localization of bleeding site. Chest roentgenography is frequently valuable in illustrating the disease process [1]. Computed tomography has also been established in recent decades as a particularly sensitive diagnostic and localizing tool in hemoptysis [2]. Endoscopic assessment, however, by rigid and/or flexible bronchoscopy is often warranted. Both a diagnostic and therapeutic tool, it is aimed to lateralize the bleeding side; localize the bleeding site and identify the cause [3,4]. In case of rapid and severe hemorrhage making airway visualization impossible, emergent rigid bronchoscopy or arteriography may be employed, for successful isolation and subsequent clogging of the bleeding vessel.

Management approach is prioritized to control ongoing hemoptysis with endotracheal intubation and intensive care monitoring. In cases with lateralized or localized persistent bleeding immediate, albeit transient, control of the airway may be obtained with topical therapy, endobronchial tamponade, or unilateral intubation of the nonbleeding lung. Angioembolization of the pathologic bleeding bronchial arterial vessels is now globally recommended as the treatment of choice in most cases [3,5]. Apart from being a definitive treatment modality, it provides valuable time and prevents bleeding during operation in patients undergoing subsequent surgical intervention. Conservative management is generally preferred and associated with lesser mortality rates [6,7]. Surgery is usually advocated in case of failure of embolotherapy, persistent hemodynamic and respiratory compromise and accurate identification of the bleeding site. Lobectomy, pneumectomy, wedge resection, segmentectomy or a combination of these approaches may be adapted. Emergent resection, however, is associated with greater risks in comparison to elective surgery [8], and should generally be reserved for when general condition and vital organs of the patient would permit surgical therapy.

Even as principal focus in critical presentation is directed towards identification and stoppage of the source of bleeding, it is the primary etiology that dictates the clinical course and treatment strategy [9,10]. Overall clinical outcome is reflected by the generalized nature of the destructive pathology and respiratory reserve. Etiology is varied and includes bronchiectasis, lung cancer, active or old tuberculosis, primary pulmonary fungal infection, lung abscess, bronchitis, pneumonia, bronchovascular fistula along with rheumatic heart disease, carcinoid and a couple of other rare causes. Together with operatibilty and rate of bleeding, these pathologies often determine the outcome. Operable patients and those with lesser rates of bleeding generally have a better prognosis [10]. Similar can be said about non-malignant causes of hemoptysis [2]. Massive hemoptysis, bleeding diathesis and lung neoplasms, however, have poorer outcomes.

## Methods/design

### Study design

It is an observational study with a cross-sectional design in which charts of all patients admitted with the presentation of haemoptysis in the past ten years will be reviewed.

### Setting

Aga Khan University Hospital, Karachi, Pakistan.

### Data collection

All patients with hemoptysis (mild, moderate or massive) will be included in this study. Prevalence is likely to vary with age. It is expected to be markedly different for pediatric age group. Only adult patients (eighteen years or older) are included in this study.

A series of variables, based on previous literature on haemoptysis related to the objectives of present study, will be determined in the study. Apart from demographics, these include smoking status, co-morbids, medications, previous hemoptysis, and the quantity of bleeding during the current episode to classify into mild, moderate or massive hemoptysis. Certain clinical features at the time of presentation like hypotension; tachycardia; tachypnea; hypoxia; bronchial breathing; wheezing, and crackles on chest auscultation, will be noted. Certain baseline investigations including levels of hemoglobin; blood urea nitrogen; creatinine and alanine transaminase, leucocytes and platelet count plus prothrombin time and activated partial thromboplastin time, will also be taken note of. Also observed will be the chest roentgenographic findings, as reported by a consultant radiologist at the institution, including presence of any infiltrates, cavity, consolidation, bronchiectasis, mass or any other significant element. Similar features of computed tomographic scan will be observed. Bronchoscopy findings included would be presence/absence of active hemoptysis and identification of bleeding site into the specific lobe.

Cause of hemoptysis would then be taken note of. In case of infectious processes the determination of etiology will be made through the microbiological analyses of culture findings (e.g. sputum culture and/or smear, in cases where culture evidence is unavailable, analysis for acid fast bacteria for identification of mycobacterium tuberculosis). For neoplastic processes, histopathological evidence will be recorded for determination of the malignancy.

The treatment modality e.g. embolotherapy, surgery or other would be marked. During of hospitalization and any complications following the management approach adapted, would be noted. Also included would be the outcome, six monthly follow-up and in case of mortality during the period, cause of death.

The above-mentioned queries would be incorporated into an observational checklist that would serve as the data collection tool.

The primary objective therefore of this study is to determine the role of various etiological agents in contributing towards hemoptysis presentation. It is hypothesized that infectious, rather than neoplastic, etiologies would contribute mainly towards hemoptysis presentation in this setting.

The other objectives include determination of the role of various investigations, i.e. chest x-ray and CT scanning, in correctly identifying the bleeding site; to determine the demographics of patients presenting with hemoptysis; and to compare the success rates of various management alternatives mentioned in stopping the bleeding.

## Results

Data analysis will be carried out by entering the data into the Microsoft Windows based Statistical Package for the Social Sciences (SPSS- released 10.1, standard version, copyright SPSS; 1989–1999). Anonymity of the subjects will be maintained. Diagnostic accuracy of various investigations will be calculated and outcome of different treatment modalities like surgery and embolization will be compared, the outcome of interest being stoppage of bleeding. Multivariate analysis will be employed.

Positive and negative predictive value of each diagnostic investigation will be calculated by assessing their ability to correctly identify the site of bleeding. Chest x-ray and CT scanning will be assessed in this manner. Also sensitivity and specificity of each investigation will be assessed by finding proportion of patients with condition who test positive, and proportion of patients without condition who test negative, respectively. For judging the ability of each investigation to discriminate between diseased and non-diseased persons, receiver operator curves may be plotted.

## Discussion

Over the world there is a marked geographical variation between the causative agents of hemoptysis. Despite the increase in HIV-associated tuberculosis in the west, studies there usually demonstrate malignancy and other non-tuberculous causes as the leading agents [2]. Developing world, largely endemic to tuberculosis, bears the brunt of the disease. Trials have shown tuberculosis to be the major source of hemoptysis in these nations [1,11]. Lacking finances and sufficient health resources, they lack indigenous studies and formal protocols on the approach to hemoptysis, based on their locoregional causative factors.

This study, generated from a third world country where tuberculosis is endemic [12], aims to obtain a thorough picture of the patterns of hemoptysis in this nation. Apart from demographics it will provide the various etiologic agents and their proportionality in contributing towards hemoptysis presentation in this country; different investigation techniques and their success rates in positive identification of the disease process; various management strategies and their results plus follow up and outcome determination.

It is the first trial of this kind from this part of the world and the results generated will contribute greatly to the knowledge and policy making of health system in not just the subcontinent, but also major parts of the world, which share similarities in disease patterns of the lung.

## Competing interests

The author(s) declare that they have no competing interests.

## Authors' contributions

Being the sole author, Omer Ashraf was involved in conception and design of study, drafting and revision of the article and in final approval of the manuscript version to be published.

## Pre-publication history

The pre-publication history for this paper can be accessed here:


